# A Four-Quadrant PVDF Transducer for Surface Acoustic Wave Detection

**DOI:** 10.3390/s120810500

**Published:** 2012-08-02

**Authors:** Zimo Lu, Dante J. Dorantes-Gonzalez, Kun Chen, Fei Yang, Baoyin Jin, Yanning Li, Zhi Chen, Xiaotang Hu

**Affiliations:** State Key Laboratory of Precision Measuring Technology and Instruments, Tianjin University, Weijin Road, No. 92, Tianjin 300072, China; E-Mails: zimo.lu@yahoo.cn (Z.L.); chenkun789@yahoo.cn (K.C.); ydishikun@163.com (F.Y.); bao420487704@163.com (B.J.); drynli@tju.edu.cn (Y.L.); ch-z@163.com (Z.C.); xthu@tju.edu.cn (X.H.)

**Keywords:** laser-induced surface acoustic wave, piezoelectric transducer, PVDF transducer, four-quadrant arrangement

## Abstract

In this paper, a polyvinylidene fluoride (PVDF) piezoelectric transducer was developed to detect laser-induced surface acoustic waves in a SiO_2_-thin film–Si-substrate structure. In order to solve the problems related to, firstly, the position of the probe, and secondly, the fact that signals at different points cannot be detected simultaneously during the detection process, a four-quadrant surface acoustic wave PVDF transducer was designed and constructed for the purpose of detecting surface acoustic waves excited by a pulse laser line source. The experimental results of the four-quadrant piezoelectric detection in comparison with the commercial nanoindentation technology were consistent, the relative error is 0.56%, and the system eliminates the piezoelectric surface wave detection direction deviation errors, improves the accuracy of the testing system by 1.30%, achieving the acquisition at the same time at different testing positions of the sample.

## 1. Introduction

Currently, nanoindentation is the main commercial measurement technique for testing the main mechanical properties of thin films. The nanoindentation method is based on the loading and unloading curve analysis of a probe at the surface of a thin film sample to obtain the elastic modulus or hardness. However, this technique still has the following shortcomings: the result is obtained indirectly, especially for unknown samples [1,2]; the lateral resolution is limited by the size of the probe [3], the reliability of the results depends on special measurement conditions and adverse influences, such as creep and thermal drift [2,4], influencing the maximum depth and the gradient of the upper unloading curve [5]; the substrate material affects the precision of the measurement greatly [6,7]; due to the surface damage inflicted by the probe, and the required previous preparation of the samples, this technology can be considered as a sort of destructive testing technique [3]. Therefore, exploring new techniques to improve the test accuracy, while reducing the sample damage is an urgent problem, and to this regard, one feasible technique is the application of polyvinylidene fluoride (PVDF) foil transducers for the detection of laser acoustic excitations in thin film surfaces.

Since PVDF is a good material for strain gauges, a sensor made of this material has good pressure and temperature change characteristics [8–10], hence, it is a common method to use PVDF piezoelectric film sensors for detecting laser ultrasonic signals [11–13]. When they are used to detect wave fronts, a pulse laser line source is more appropriate between the sensor and the probe [14], and the probe should be placed exactly on the direction of the surface acoustic wave propagation [15], however there is not yet a method to adjust the probe precisely.

The current investigation proposes an alternative to solve this problem by designing a novel piezoelectric sensor, which has four-quadrants testing parts, since they look like the four-quadrants divided by the two ordinate and abscissa axes, so we called it four-quadrant piezoelectric sensor, this sensor can test whether the probe is placed in the correct direction of the calibration, in addition, the experimental dispersion curve of surface acoustic wave is calculated, for what, signals at two different detecting points are needed. The traditional PVDF piezoelectric detection systems require moving the probe from one point to the second testing point, thus, twice introducing measurement repetition errors. The experimental design of the four-quadrant piezoelectric acoustic surface wave probe can eliminate this error, and achieve real-time acquisition of signals in different detection locations.

## 2. Surface Acoustic Wave Excitation Patterns and Directivity

Surface acoustic waves have aroused attention as an effective nondestructive testing technique. Based on the surface acoustic wave propagation along the surface, surface waves have an intrinsic interaction with the material surface, so this technique is an ideal method to detect the mechanical properties of near surface layers [15].

**Figure 1.**
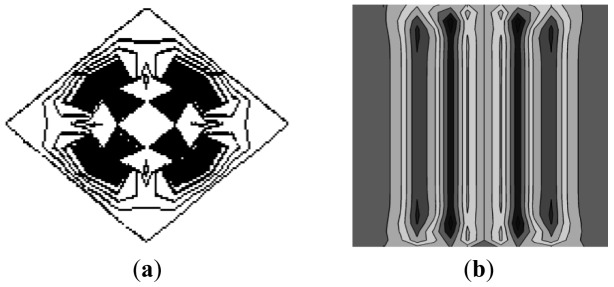
Wave front of SAW on Si sample: (**a**) wave front excited by a point source, (**b**) wave front excited by a line source.

Laser excitation is mainly divided into point source and line source excitation. Figure 1 shows the simulation of surface acoustic wave fronts propagation. Figure 1(a) shows a wave front flat pattern generated by a point laser source. From the figure, it could be seen that the wave front pattern is irregular, and if a linear wedge piezoelectric pressure probe is used to detect the point-source-generated surface acoustic wave, the detection line inevitably falls in different phase wave fronts, leading to detection signal loss of its original physical meaning [16]. Figure 1(b) shows a laser beam line source exciting a surface acoustic wave front flat pattern. As shown, the wave front is a somewhat regular straight line with a typical wave front flat pattern, hence indicating that using a linear wedge with a straight line to detect surface acoustic wave is suitable, and that's why SAW testing experimental systems usually use line source to stimulate the surface acoustic wave fronts [16].

After selecting the line source to generate SAW, by only ensuring that the detection line is perpendicular to the surface acoustic wave propagation direction, the detection signals could be ensured to have the same phase. To solve the probe placement directivity and real-time measurement problems, this experiment proposed the design of a four-quadrant piezoelectric sensor.

## 3. The Working Principle and Design of a Four-Quadrant Piezoelectric Sensor

This experiment utilizes a PVDF piezoelectric sensor to detect surface acoustic waves in thin films. PVDF has unique dielectric, piezoelectric and thermal effects, so it is capable of converting mechanical signals into electrical signals; and its output electric charge is proportional to the stress perpendicular to the surface [16].

**Figure 2.**
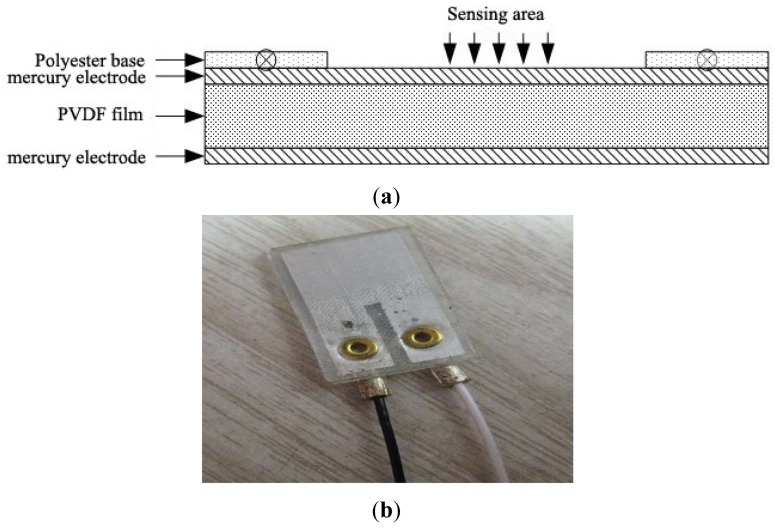
PVDF piezoelectric sensor: (**a**) schematic structure of PVDF foil piezoelectric transducer; (**b**) PVDF foil piezoelectric transducer physical picture.

**Figure 3.**
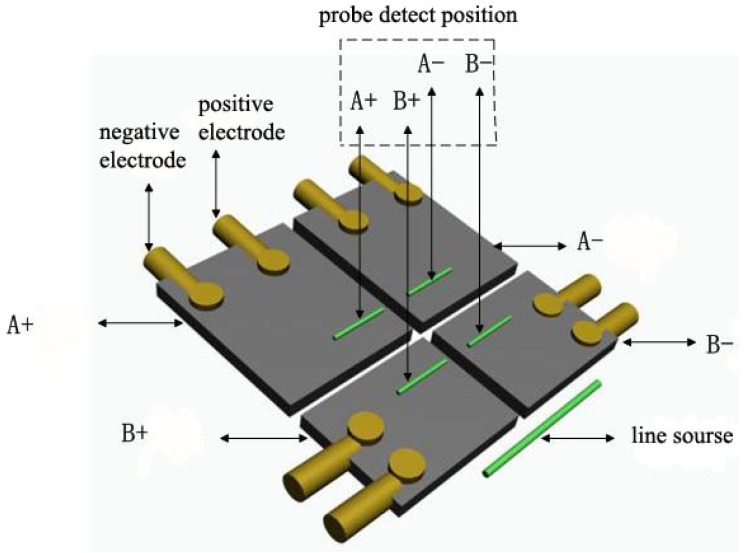
Four-quadrant PVDF foil piezoelectric SAW transducer.

The piezoelectric sensor is composed of a piezoelectric material PVDF foil with both positive and negative sides, coated with a silver electrode. To convert this material into a surface acoustic wave piezoelectric transducer device, hydrofluoric acid (HF) was used to corrode and form electrode patterns, as well as used copper rivets to connect the two electrode leads. A schematic figure and a picture of the PVDF foil piezoelectric transducer structure are shown in Figure 2. On the basis of this basic PVDF foil transducer and harnessing the properties of the foil to meet the demands of the probe directivity, a combined structure of the PVDF foil transducer as a four-quadrant piezoelectric transducer was designed, and shown in Figure 3.

**Figure 4.**
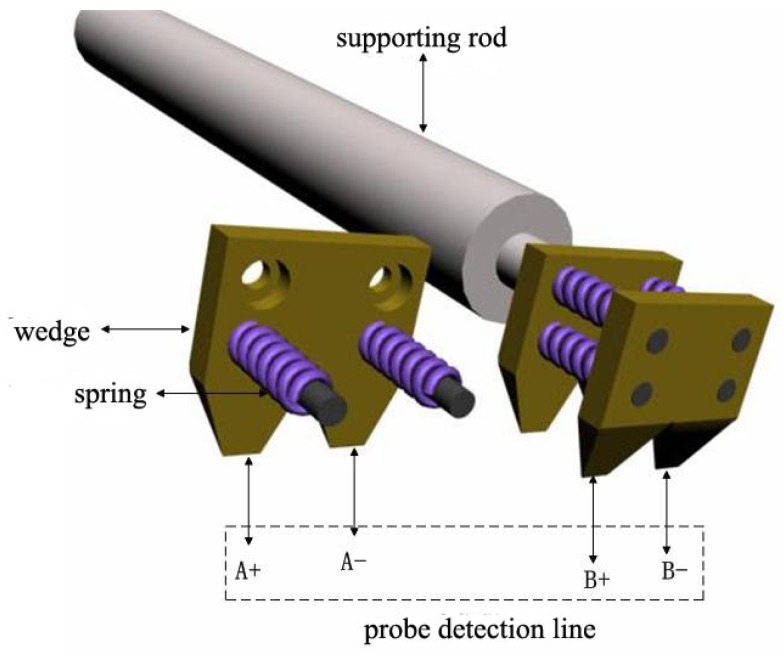
Four-quadrant wedge probes.

Based on the propagation theory of line sources for exciting surface acoustic waves, the detection region was divided into four quadrants A+, A−, B+, and B−. There are four quadrant piezoelectric foil transducers (see Figure 3) and four quadrant wedges (see Figure 4) on the corresponding locations respectively. When the signals obtained by A+ and A− coincide, as well as the signals obtained by B+ and B− coincide, that indicates that the detection line is paralleled with the wave front, and meaning that the detection line is perpendicular to the surface acoustic wave propagation direction. Otherwise, the detection line deviates from the surface acoustic wave front. Adjustment for the position of the four-quadrant wedges is needed until the signals from the corresponding quadrants coincide. Signals A+ and B+ or signals of A− and B− are used to calculate the dispersion curves, and what is more, the dispersion curves are calculated from the same excitation pulses picked up in different locations at the same time.

Hydrofluoric acid (HF) was used to corrode four mutually insulated sensing quadrants on the PVDF foil surface and two electrodes “+” “−” in each quadrant. Wires were connected to the electrodes by clips and copper rivets. During the experiment, the four-quadrant wedge probes and the excitation line sources are posited as shown in Figure 3. The calibration of the SAW propagation direction was achieved by adjusting the orientation of the probe.

In the four-quadrant wedge probe showed in Figure 4, each probe arrangement includes two basic symmetry metal bodies, and each metal body contains two wedges, with a gap of 2 mm between the two wedges. Both metal bodies have four ring-shaped grooves on them, and four screws surrounded by springs, tighten the metal bodies, regulating the distance between the two main metal bodies, which is the distance between the two testing points on the direction of the propagated SAW. This design can adjust not only the perpendicularity of the probe to the wave propagation direction, but also adjust the distance between the two detection probing points, hence reaching a two-dimensional adjustment, ensuring SAW signal measurements accurately, and detecting the signals at different locations simultaneously, hence eliminating the direction deviation and the repeatability errors of measurement.

## 4. The Experimental Setup and Calculation Principle

**Figure 5.**
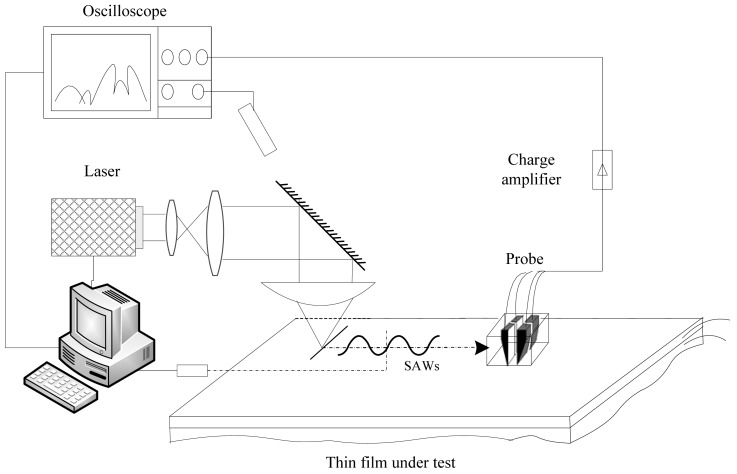
PVDF piezoelectric transducer SAW detection system schematic diagram.

The system used a 532 nm wavelength, 800 ps pulse laser as the surface acoustic wave excitation source. The pulse laser passes through a collimating and expanding beam treatment, it is reflected downwards, and passed through a cylindrical convex lens, gathering the beam into a straight line on the surface of the sample, and consequently, generating the SAW. After the surface waves propagate along the sample surface for a certain distance, they are detected by an oscilloscope acquisition system, passing through a charge amplifier, and then the experimental signals are loaded into a computer that process and uses an algorithm that compares the dispersion curve with that of a theoretical model, thereby obtaining the Young's modulus of the thin film. A schematic diagram of the SAW detection system is shown in Figure 5.

## 5. Experimental Results

In this paper, the design of a four-quadrant piezoelectric transducer SAW detection system improves the sensing capability of an existing simple piezoelectric SAW detection system, since now the probe direction can be calibrated by the time domain waveforms, hence eliminating the error introduced by the direction of the propagation signal, and besides, measuring two SAW signals at two different detection points at the same time. The experimental sample is a SiO_2_ thin layer on Si substrate. Specific physical parameters of the sample are shown in Table 1.

**Figure 6.**
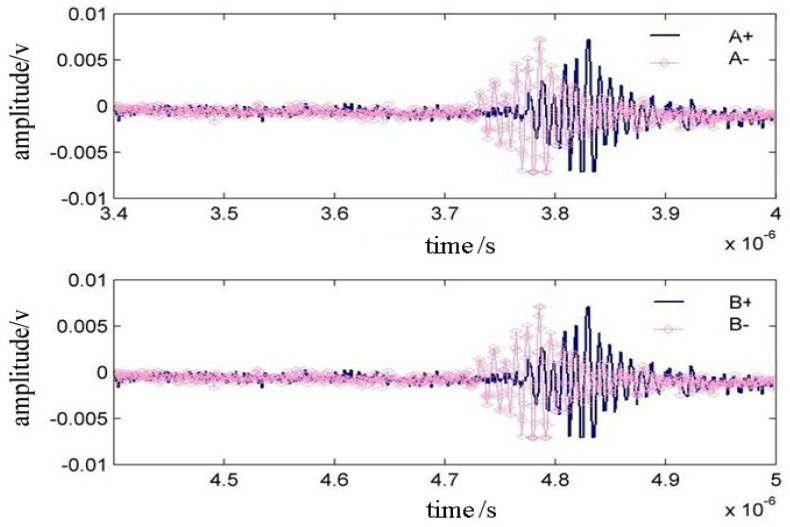
Waveform in time domain when the detecting line deviates 1 degree.

In the experiment, when the four-quadrant piezoelectric probe direction and the surface acoustic wave propagation direction have a small angle deviation, the output signal of the four-quadrant probe has the following performance as shown in Figure 6. At this point, the time domain A+ and A− waveforms have an obvious deviation compared with the time domain B+ and B− waveforms, indicating that an adjustment of the direction piezoelectric probe is needed.

**Figure 7.**
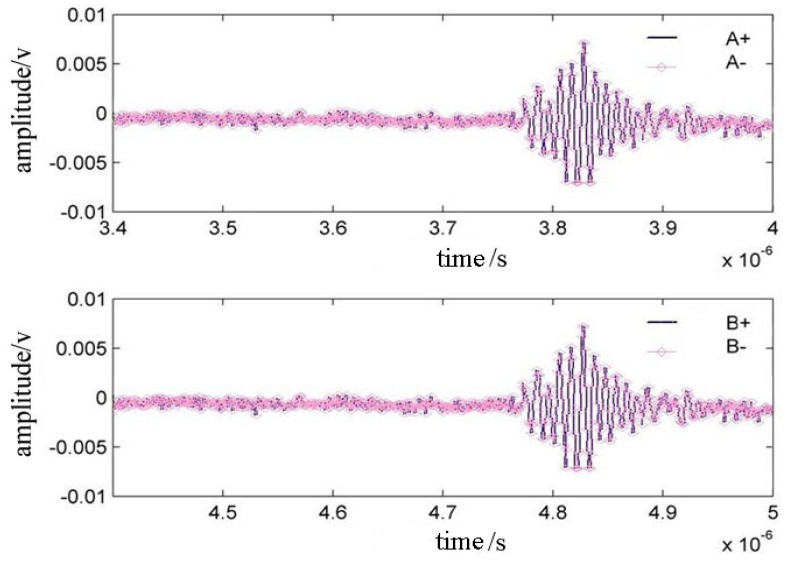
Overlapped waveforms in time domain when the detecting line coincides with the propagating direction.

By adjusting the orientation of the probe, we can make the time domain A+ and A− waveforms to overlap, as well as the time domain B + and B− waveforms as shown in Figure 7.

However, there may be other situations when A+/A− waveforms and B+/B− waveforms could be different, for instance, no matter how accurate we adjust the angle between the sensor and the sample, the waveforms still cannot overlap each other, then this phenomenon shows us that some deviations caused from material properties discrepancy and/or fabrication inaccuracies, as an additional result, are being encountered. Our recommendation in this case is to move the sensor to other sample region in order to investigate the sample homogeneity and a proper region to calculate the Young's modulus.

After the adjustment of the probe is made, then we can ensure a more reliable calculation of the experimental dispersion curve from the acquired surface acoustic waves, and compare with the theoretical dispersive curves.

The calculation procedure to determine the Young's modulus of a thin film is the following. First we compute the experimental relation of the SAW phase velocity propagating in the sample *versus* the working frequency range, the so called dispersion curve, by using Equation (1):

(1) $$c(f)=\frac{2\pi f(d_{2}-d_{1})}{\Phi_{2}(f)-\Phi_{1}(f)}$$

where *f* is the elastic wave frequency, *d_1_* and *d_2_* are two different testing distances detected along the A+/B+ and A−/B− directions perpendicular to the laser beam line. *Φ_1_(f)* and *Φ_2_(f)* are the phase spectra of the two sampling points respectively, they can be obtained by Fourier transforming the surface wave impulses received at A+/B+ or A−/B−. Second, we compute a theoretical set of dispersion curves by using surface wave propagation in elastic media, *c* = *c*(*E, E′, v, v′, ρ, ρ′, d*), where *E* and *E*′ are the Young's modulus, *v* and *v*′ are the Poisson ratios, *ρ* and *ρ*′ are the densities and *d* is the film thickness. By fixing the values of thickness, density and Poisson ratio, and for a series of known values of Young's modulus, we obtain a set of theoretical dispersion curves. Finally, we fit the experimental dispersion curve to match the nearest curve from the set of theoretical dispersion curves by a least squares method. The Young's modulus of the best matched theoretical dispersion curve represents the Young's modulus of the tested thin film.

**Figure 8.**
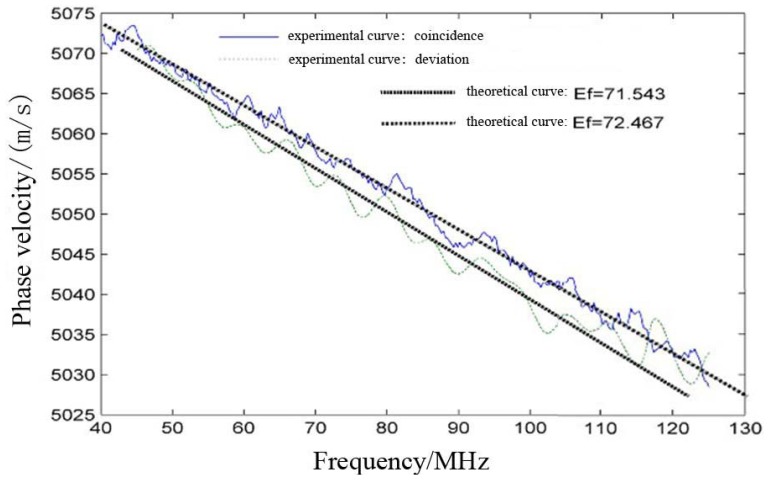
Dispersion curve fitting results.

In Figure 8 we can observe the fitting results of the theoretical curves with, first, an experimental dispersion curve under coincidence conditions of the probe and, second, with an experimental dispersion curve without adjusting the coincidence of the probe with the propagation direction. As one can see from Figure 8, the final results of the fitting procedure for the two experimental dispersion curves with adjustment and without adjustment can turn into different Young's modulus values, as in this case, 72.467 6 and 71.543 GPa, respectively, meaning an improvement of 1.3% in relative error.

**Figure 9.**
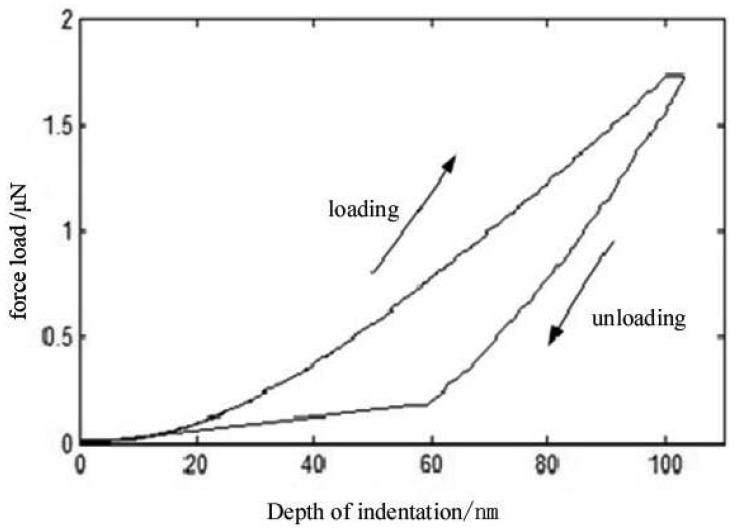
Nanoindentation loading and unloading curves.

Since nanoindentation is the main reliable commercial measurement method to detect Young's modulus of thin films, a cross validation of this results may be useful for experimental verification purposes. Therefore, a commercial nanoindentation equipment from the Nano company was used to test the Young's modulus of the sample, resulting in the loading and unloading curves shown in Figure 9.

Based on the analysis of unloading curve, the value of Young's modulus of this sample was found to be 71.147 GPa. As shown in Table 2, the results of the four-quadrant piezoelectric transducer detection method and the nanoindentation measurements are basically consistent. The relative error is 0.56% when the piezoelectric probe is adjusted, while when the probe has 1° deviation, the relative error in comparison with nanoindentation is 1.86%. From these results, we could demonstrate that the four-quadrant piezoelectric foil probe measurement is consistent and accurate, and the acquisition of the surface waves can be tested in two different locations at the same time.

## 6. Conclusions

In this paper, a novel four-quadrant piezoelectric foil transducer was designed and used for consistently detecting laser-induced surface acoustic waves. This improvement achieved a better adjustment of the probe detection direction with respect to the propagation direction, so that the accuracy of measurement is improved by 1.30% in comparison with a 1° deviated probe, while the probe also was able to detect two surface wave signals at two different testing points simultaneously, thus eliminating the direction deviation error.

## Figures and Tables

**Table 1.** Physical parameters of thermal oxidation of SiO_2_/Si samples.

	**SiO_2_ film**	**Si substrate**
density (kg/m^3^)	1,300	2,300
Poisson's ratio	0.26	0.27
Elastic constant (10^10^ N/m^2^)	c_11_, c_12_, c_13_, c_22_, c_44_	c_11_ = 16.57, c_12_ = 6.39, c_44_ = 7.956
Film thickness (nm)	230	–
Young's modulus (GPa)	To be measured	160

**Table 2.** The comparison between the results of the nanoindentation and the four-quadrant piezoelectric detection techniques.

	**Nanoindentation**	**Piezoelectric detection with adjusted probe**	**Piezoelectric detection with the probe deviated 1°**
Young's modulus value for the SiO_2_/Si sample	71.147 GPa	71.543 GPa	72.46 7 GPa
Relative error (%)	–	0.56	1.86
